# Environmental Factors Associated with Growth and Major Compound Accumulation in *Dendropanax trifidus*

**DOI:** 10.3390/plants15213415

**Published:** 2026-11-03

**Authors:** Dong Hwan Lee, Jun Hyuk Jang, Ji Ah Kim

**Affiliations:** 1 Forest Medicinal Resources Research Center, National Institute of Forest Science, Yeongju-si 36040, Republic of Koreawnseldu123@korea.kr (J.H.J.); 2 Department of Forest Bioresources, National Institute of Forest Science, 39 Onjeong-ro, Gwonseon-gu, Suwon 16631, Republic of Korea

**Keywords:** *Dendropanax trifidus*, UPLC, medicinal plants, correlation, secondary metabolites

## Abstract

*Dendropanax trifidus* is an evergreen broad-leaved tree valued for its bioactive compounds, particularly chlorogenic acid (CGA) and rutin. This study investigated the relationships among growth characteristics, major compound contents, and environmental conditions in *D. trifidus* leaves collected from 17 sites (14 cultivated sites and three natural habitat) in southern Korea and Jeju Island. A total of 67 individual trees were sampled, and growth traits, CGA and rutin contents, soil physicochemical properties, and meteorological variables were evaluated. Pearson correlation analysis and principal component analysis (PCA) were performed using site-level mean values (*n* = 17) to account for the hierarchical sampling structure. Rutin content generally exceeded CGA content, with combined compound contents ranging from 0.27 to 24.17 mg/g DW across the 17 collection sites. In contrast, the three natural populations at elevations of 308–535 m exhibited relatively higher CGA than rutin contents. PCA revealed contrasting multivariate patterns among major compound contents, growth characteristics, and environmental variables, with the first two principal components explaining 51.43% of the total variance. Following false discovery rate (FDR) correction, tree height and number of lobes were positively correlated with altitude, while the dry weight of 10 leaves was positively correlated with soil available phosphorus. However, no significant associations between major compound contents and growth or environmental variables were identified after FDR correction. These findings provide preliminary insights into variation in the growth and phytochemical characteristics of *D. trifidus* across different environments and may inform future investigations aimed at identifying suitable cultivation conditions for targeted compound production. Further studies involving more sampling sites and controlled environmental conditions are needed to validate these relationships.

## 1. Introduction

The genus *Dendropanax*, a member of the family *Araliaceae*, comprises approximately 97 species. Among them, *Dendropanax trifidus* (Thunb.) Makino ex H.Hara is a subtropical evergreen broad-leaved tree distributed across Korea, Japan, the Nansei Islands, and Taiwan [1,2]. *Dendropanax trifidus* was previously distinguished from *D. morbiferus*; however, both morphological [3] and genetic evidence [4] have indicated insufficient differentiation to clearly support their treatment as separate species. Consequently, the two taxa are currently regarded as a single species [5]. The species has traditionally been valued for its distinctive golden-colored sap, which has been used as a high-quality natural coating material [6]. It has also been used in traditional medicine for various purposes, including the treatment of inflammation, menstrual irregularities, and migraines, as well as for improving blood circulation [7].

Research on the biological activities of *D. trifidus* was initially limited, with early reports including its potential to induce allergic contact dermatitis [8,9]. More recently, however, a growing body of research has demonstrated a broad range of pharmacological activities associated with different plant parts. The sap of *D. trifidus* has been reported to exhibit hypoglycemic effects, suppress weight gain, and enhance energy metabolism in muscle cells [10,11]. Extracts obtained from the leaves, branches, bark, and stems have also demonstrated antioxidant, antidiabetic, anti-inflammatory, and immunomodulatory properties [12,13,14,15]. These biological activities are likely associated with the diverse array of secondary metabolites found within the genus *Dendropanax*, including polyphenols, flavonoids, tannins, pyrimidines, essential oils, terpenoids, phenolic acids, and alkaloids [16]. Accordingly, *Dendropanax* species have attracted considerable attention as potential sources of bioactive natural products for applications in the pharmaceutical, food, and cosmetic industries.

Plant secondary metabolites play important roles in adaptation to environmental conditions and protection against biotic and abiotic stresses. For instance, flavonoids can contribute to the protection of cellular components from ultraviolet radiation, whereas tannins can deter herbivory through their characteristic astringency [17]. Beyond their ecological functions, these metabolites are also important determinants of the biological activities and medicinal value of plants. Consequently, both the quantity and composition of secondary metabolites are critical factors in assessing the quality and potential utilization of medicinal plant resources. Despite the increasing recognition of the medicinal potential of *D. trifidus*, relatively little is known about how geographical variation and environmental conditions, particularly climatic and soil factors, are associated with the accumulation of major phenolic compounds such as chlorogenic acid (CGA) and rutin in its leaves. In particular, the relationships between these compounds and environmental or plant growth characteristics across different growing environments remain insufficiently understood. Such information is important not only for understanding the ecological and chemical variation in the species but also for informing future investigations into cultivation conditions associated with desirable phytochemical profiles.

Previous studies have demonstrated that environmental conditions are associated with variation in secondary metabolite accumulation in medicinal plants. For example, geographical and climatic factors have been associated with differences in bioactive compound profiles in *Dendropanax dentiger*, a species belonging to the same genus as *D. trifidus* [18]. In a previous study, Son et al. (2025) [19] investigated the relationships among environmental conditions, growth characteristics, and secondary metabolite accumulation in *Eleutherococcus sessiliflorus*, another member of the *Araliaceae* family, across cultivation sites in South Korea. Research on *Aralia elata* has also highlighted the potential influence of light availability and soil nutrient conditions on phenolic-based chemical traits [20]. Collectively, these studies suggest that environmental conditions may be associated with variation in plant growth and secondary metabolite accumulation, although the relationships may differ among species and compounds. A better understanding of the relationships among environmental conditions, plant growth, and secondary metabolite accumulation in *D. trifidus* could therefore contribute to the development of strategies for selecting suitable cultivation sites and producing leaves with desirable phytochemical profiles.

Accordingly, this study investigated the relationships between environmental conditions, growth characteristics, and the accumulation of major compounds in *D. trifidus* populations. Field investigations were conducted at 17 cultivated and natural populations across southern Korea and Jeju Island to identify environmental factors associated with variation in plant growth and the accumulation of CGA and rutin. Specifically, this study examined whether CGA and rutin differed in their associations with climatic, soil, and plant growth variables, and whether phytochemical profiles varied across different habitat conditions. By addressing these questions, this study aims to improve our understanding of environmental variation in the growth and phytochemical characteristics of *D. trifidus* and provide preliminary information relevant to future studies on cultivation site selection and the production of leaves with desirable phytochemical profiles.

## 2. Results

### 2.1. Growth Characteristics of D. trifidus

**Figure 1:**
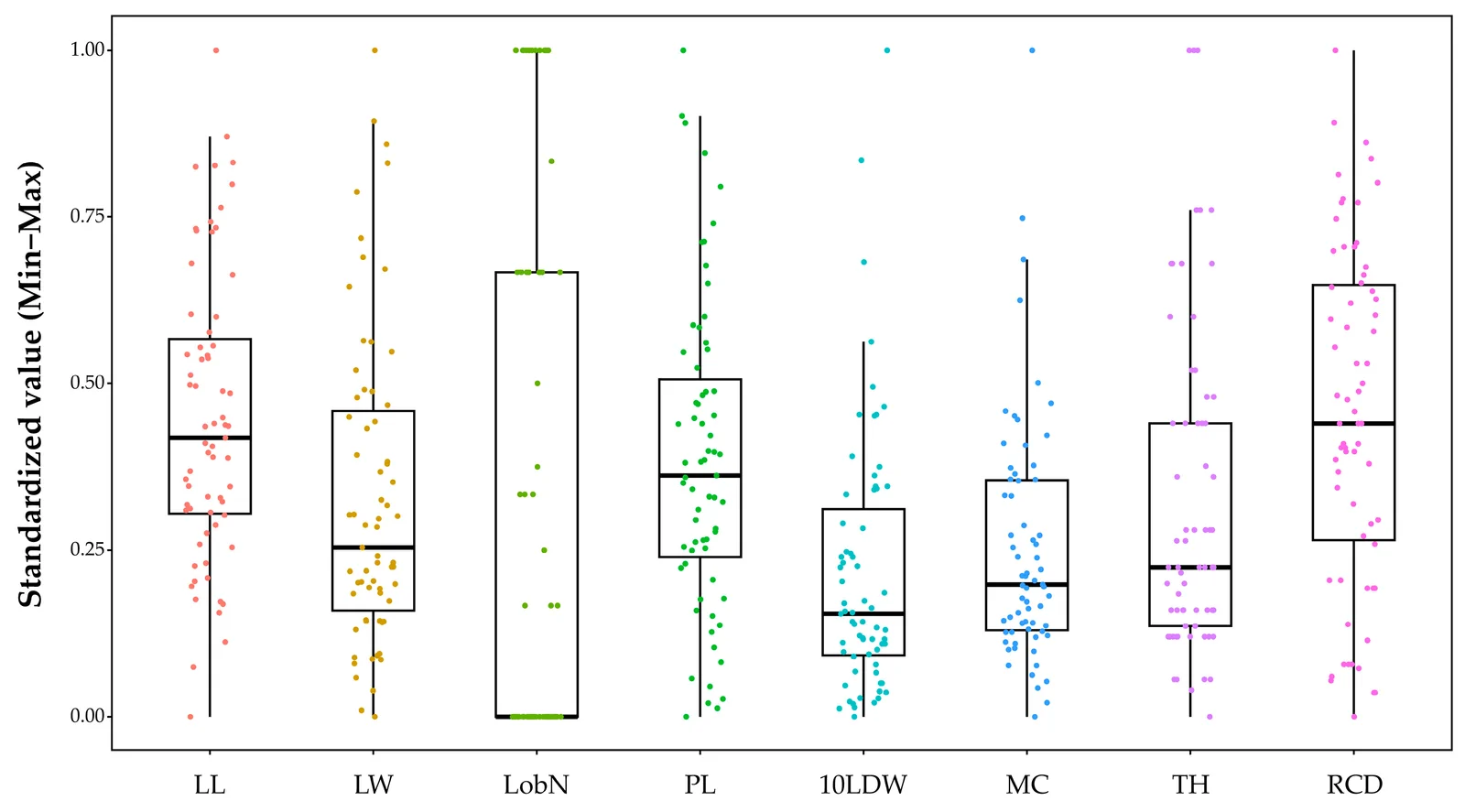
Distribution of standardized growth characteristics among the collected samples of *Dendropanax trifidus*. LL, leaf length; LW, leaf width; LobN, number of lobes; PL, petiole length; 10LDW, 10 leaves dry weight; MC, moisture contents; TH, tree height; RCD, root collar diameter.

Figure 1 and Table 1 summarize the growth characteristics of *D. trifidus* across the 17 collection sites. Most growth characteristics showed considerable site-to-site variation. Leaf length (LL) ranged from 115.68 ± 7.22 to 160.96 ± 5.17 mm, while leaf width (LW) varied more widely, from 72.80 ± 3.82 to 154.81 ± 12.17 mm. The number of lobes (LobN) varied from 0 to 2, and petiole length (PL) ranged from 71.71 ± 9.06 to 133.52 ± 10.52 mm. Similarly, dry weight of 10 leaves (10LDW) ranged from 4.65 ± 0.52 to 15.33 ± 1.76 g, indicating substantial differences in leaf biomass among sites. The moisture content (MC) varied from 27.75 ± 4.47 to 36.90 ± 1.11%.

Tree size also differed markedly among the collection sites. TH ranged from 2.40 ± 0.20 to 11.67 ± 1.21 m, whereas root collar diameter (RCD) ranged from 4.15 ± 0.15 to 15.75 ± 0.88 cm. In particular, the broad ranges observed for LW, 10LDW, TH, and RCD indicate pronounced variation in both leaf morphology and overall tree size among collection sites. The site-specific differences were also reflected in the distribution of standardized growth characteristics shown in Figure 1. Tree age was not determined in this study.

### 2.2. Environmental Data of Collecting Sites

The physicochemical properties of soils collected from 14 cultivated sites and three natural habitats of *D. trifidus* were analyzed (Tables S1 and S2). Soil texture was classified as clay loam at all collection sites. Soil pH ranged from 4.26 ± 0.03 to 6.07 ± 0.26, indicating predominantly acidic soil conditions. Electrical conductivity (EC) and organic matter (OM) showed substantial variation among sites, ranging from 0.78 ± 0.10 to 2.81 ± 0.20 dS/m and from 2.14 ± 0.42 to 39.61 ± 0.96%, respectively. Total nitrogen (TN) ranged from 0.12 ± 0.01 to 1.10 ± 0.06%, while available phosphate (AP) ranged from 55.08 ± 11.49 to 919.16 ± 199.44 mg/kg. Cation exchange capacity (CEC) ranged from 12.68 ± 0.49 to 58.02 ± 4.78 cmol^+^/kg.

Nine meteorological variables were evaluated for the collection sites (Table S3). Annual average temperature (AAT) ranged from 15.0 to 18.3 °C, while annual average maximum temperature (AAMT) and annual average minimum temperature (AAmT) ranged from 19.5 to 21.7 °C and 10.3 to 15.5 °C, respectively. The annual maximum temperature (AMT) reached 37.0 °C at site 6, whereas the lowest annual minimum temperature (AmT) was −8.4 °C at site 7. Total precipitation (TP) ranged from 1136.1 to 2455.3 mm, and relative humidity (RH) ranged from 67.5 to 79.3%. Sunshine duration (SSH) varied from 1754.8 to 2258.8 h, while altitude (ALT) ranged from 10.0 to 535.4 m.

### 2.3. Validation and Quantification of Major Compounds

**Figure 2:**
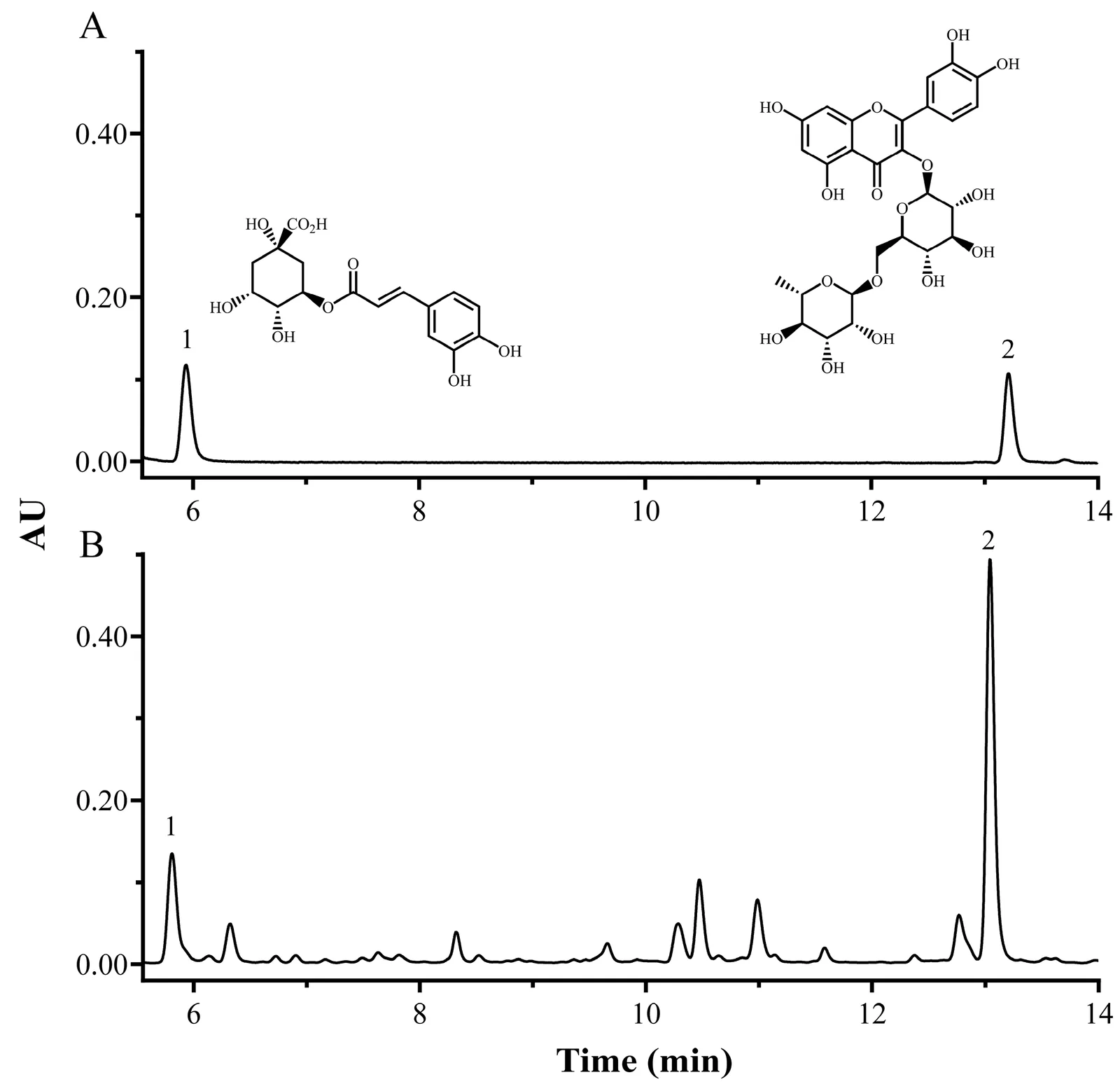
Ultra-performance liquid chromatography (UPLC) chromatogram of major compounds of the standard mixture (**A**) and *Dendropanax trifidus* leaf sample (**B**). 1, Chlorogenic acid; 2, Rutin. Chromatograms were re-plotted using GraphPad Prism 8.

The UPLC analytical methods developed for the quantification of the major compounds were validated, and the results are presented in Table 2 and Table 3 and Figure 2. Two major compounds were identified by comparing the retention times of compounds in *D. trifidus* leaf extracts with those of authentic reference standards. The calibration curves for both compounds showed excellent linearity, with correlation coefficients (R^2^) greater than 0.9999.

The limit of detection (LOD) and limit of quantification (LOQ) for chlorogenic acid (CGA) were 0.49 and 1.50 µg/mL, respectively, whereas those for rutin were 0.03 and 0.10 µg/mL, respectively. Method precision was evaluated at three concentration levels using three replicate measurements within a single day (intra-day precision) and on different days (inter-day precision). Relative standard deviation (RSD) values ranged from 0.15 to 0.26% for intra-day precision and from 0.05 to 0.30% for inter-day precision. These low RSD values demonstrated good repeatability and reproducibility of the analytical method. Overall, the validation results confirmed that the developed UPLC method was suitable for the reliable quantification of CGA and rutin in *D. trifidus* leaves.

**Figure 3:**
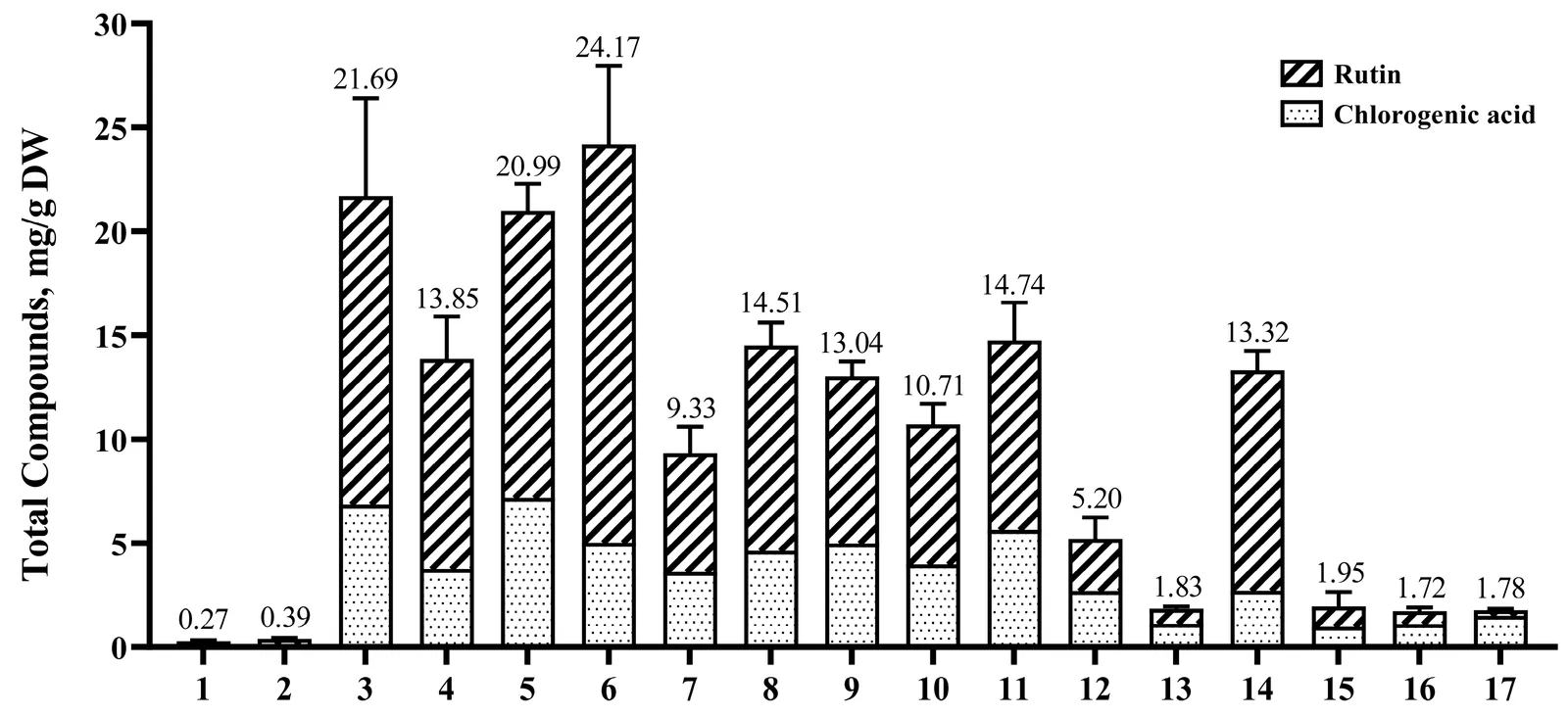
Contents of two major compounds in *Dendropanax trifidus* leaves by cultivation and habitat sites. Data are presented as mean ± standard error (SE) based on four biological replicates per site, except at one site with three biological replicates. The graph was generated using GraphPad Prism 8.

CGA and rutin were subsequently quantified in 67 leaf samples collected from 17 sites (Figure 3 and Table S4). Considerable variation in the concentrations of both compounds was observed among the collection sites, with rutin generally occurring at higher concentrations than CGA. CGA concentrations ranged from 0.08 ± 0.01 mg/g DW at site 1 to 7.17 ± 0.57 mg/g at site 5, whereas rutin concentrations ranged from 0.20 ± 0.06 mg/g at site 1 to 19.16 ± 3.79 mg/g at site 6. The combined concentration of CGA and rutin ranged from 0.27 ± 0.06 to 24.17 ± 4.31 mg/g, with the highest total concentration observed at site 6.

### 2.4. Correlation Among Compounds, Growth, and Environments

**Figure 4:**
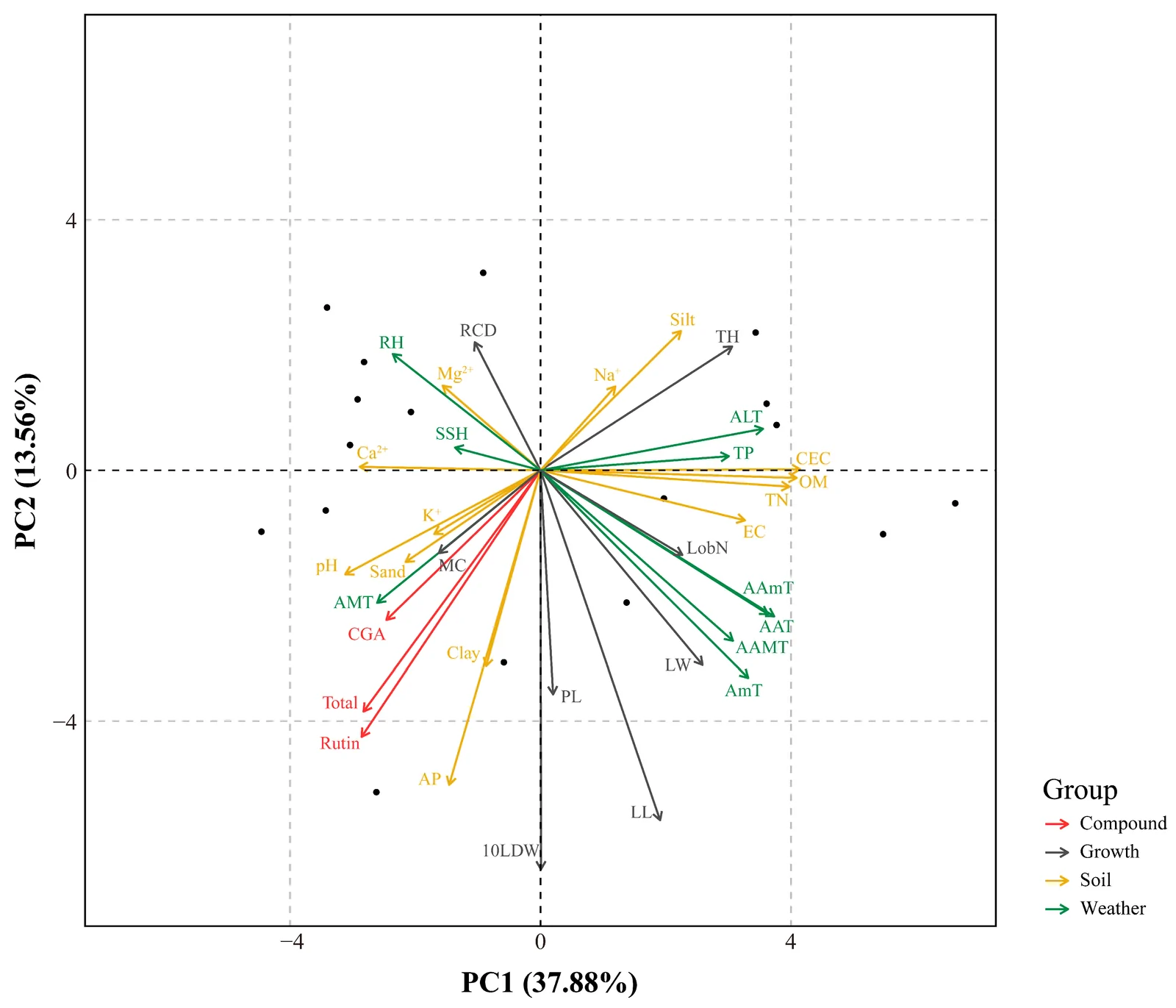
Principal component analysis (PCA) plot of the relationships among environmental variables, growth traits, and compounds in *Dendropanax trifidus*. Red arrow, compounds; gray arrow, growth traits; yellow arrow, soil properties; green arrow, meteorological factors. Each point represents an individual sample in the PCA score plot. CGA, chlorogenic acid; LL, leaf length; LW, leaf width; LobN, number of lobes; PL, petiole length; 10LDW, 10 leaves dry weight; MC, moisture contents; TH, tree height; RCD, root collar diameter; EC, electrical conductivity; OM, organic matter; TN, total nitrogen; AP, available phosphate; CEC, cation exchange capacity; AAT, annual average temperature; AAMT, annual average maximum temperature; AAmT, annual average minimum temperature; AMT, annual maximum temperature; AmT, annual minimum temperature; TP, total precipitation; RH, relative humidity; SSH, sunshine hours; ALT, altitude.

Principal component analysis (PCA) was performed using site-level mean values from 17 sampling sites to explore the multivariate relationships among major compound contents, growth characteristics, soil physicochemical properties, and meteorological variables (Figure 4 and Table S5). The first two principal components explained 51.43% of the total variance, with PC1 accounting for 37.88% and PC2 for 13.56%. The PCA biplot revealed contrasting patterns between major compound contents and several environmental and growth variables. CGA, rutin, and total compound content were positioned in the negative direction of PC1, whereas ALT, soil OM, CEC, TN, and TH were positioned in the positive direction. Several temperature-related variables were also oriented toward the positive side of PC1. CGA, rutin, and total compound content showed similar vector orientations, indicating broadly consistent patterns of variation in the PCA space. Along PC2, TH and RCD were oriented toward the positive direction, whereas LL, LW, and 10LDW were oriented toward the negative direction. These patterns suggest differences in the multivariate associations of tree growth and leaf characteristics with environmental variables. Overall, the PCA provided an exploratory overview of the relationships among phytochemical contents, plant growth, and environmental conditions across the sampled sites.

**Figure 5:**
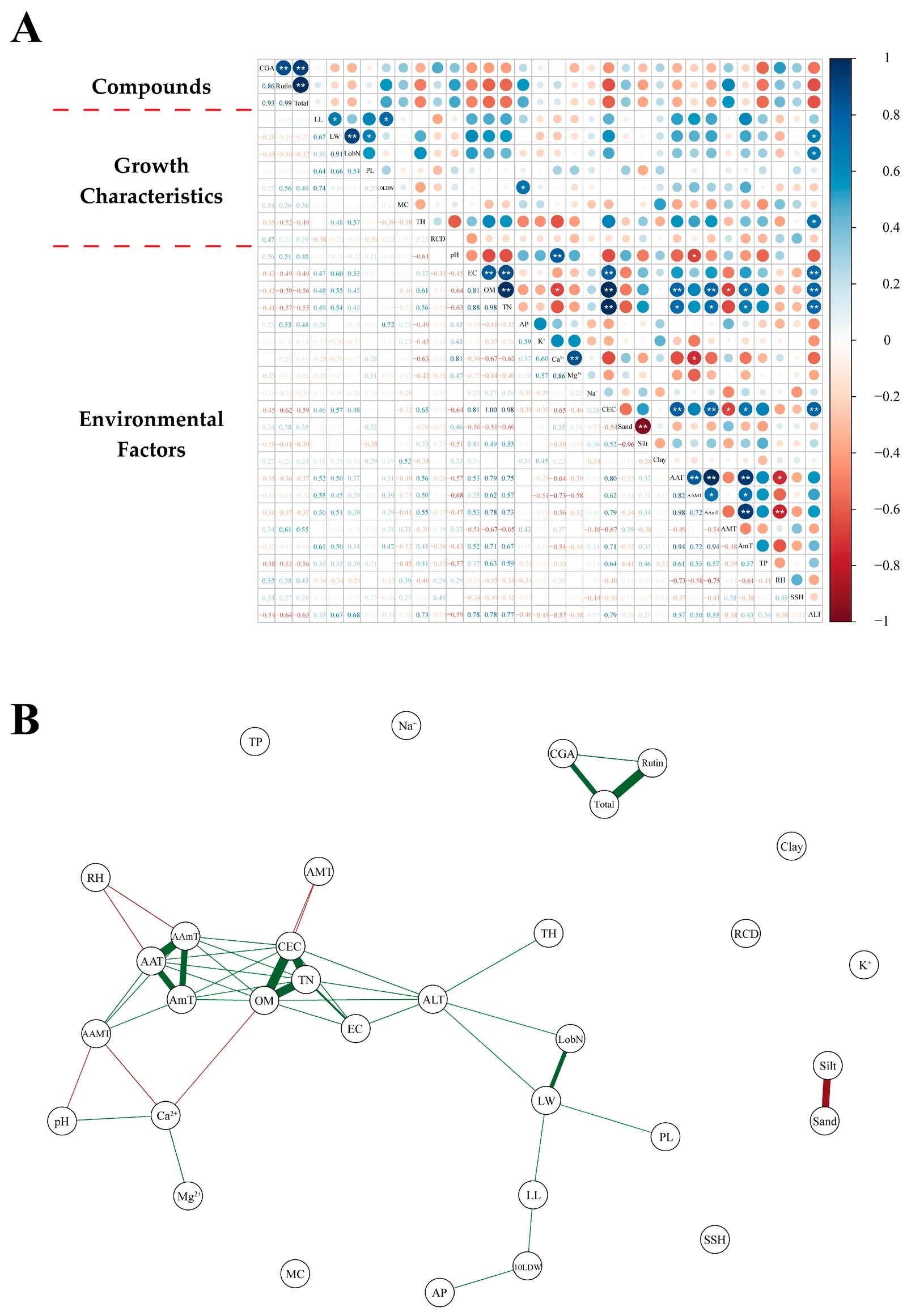
Correlation heatmap and network analysis of major compounds, growth characteristics, and environmental variables. (**A**) Pearson’s correlation matrix heatmap and (**B**) network visualization of statistically significant correlations after FDR correction. ** FDR-adjusted *p* < 0.01, * FDR-adjusted *p* < 0.05; green lines indicate positive correlations, and red lines indicate negative correlations. CGA, chlorogenic acid; LL, leaf length; LW, leaf width; LobN, number of lobes; PL, petiole length; 10LDW, 10 leaves dry weight; MC, moisture contents; TH, tree height; RCD, root collar diameter; EC, electrical conductivity; OM, organic matter; TN, total nitrogen; AP, available phosphate; CEC, cation exchange capacity; AAT, annual average temperature; AAMT, annual average maximum temperature; AAmT, annual average minimum temperature; AMT, annual maximum temperature; AmT, annual minimum temperature; TP, total precipitation; RH, relative humidity; SSH, sunshine hours; ALT, altitude.

Pearson’s correlation analysis was performed using site-level mean values (*n* = 17) to investigate the relationships among growth characteristics, major compound contents, and environmental variables (Figure 5 and Tables S6–S8). To account for multiple comparisons, *p*-values were adjusted using the Benjamini–Hochberg false discovery rate (FDR) procedure. The correlation matrix (Figure 5A) and network analysis (Figure 5B) revealed significant associations primarily among environmental variables and selected growth characteristics, whereas associations between major compound contents and growth or environmental variables were not statistically significant after FDR correction. Among the growth characteristics, the 10LDW was positively correlated with AP (*r* = 0.724, adjusted *p* = 0.016), while the LobN was positively correlated with ALT (*r* = 0.681, adjusted *p* = 0.036). TH was also positively correlated with ALT (*r* = 0.726, adjusted *p* = 0.016). Significant positive correlations were observed among several soil properties, particularly OM, TN, and CEC, as well as among temperature-related meteorological variables. These relationships were reflected in the correlation network, which showed interconnected groups of environmental variables and selected growth characteristics.

Among the major compounds, CGA, rutin, and their total content showed significant positive correlations with one another, forming a distinct group in the correlation network. Although CGA and rutin exhibited positive or negative correlation coefficients with several growth and environmental variables, none of these associations remained statistically significant after FDR correction (Table S6 and Figure 5A). Accordingly, no significant connections between major compound contents and growth or environmental variables were identified in the correlation network (Figure 5B). Overall, the results indicated significant associations among several environmental and growth variables, while the relationships between major compound accumulation and the measured environmental or growth characteristics remained statistically inconclusive at the site level.

## 3. Discussion

Considerable variation in plant growth and major compound accumulation was observed among the *Dendropanax trifidus* populations examined in this study. *D. trifidus* is an evergreen broad-leaved tree distributed mainly in relatively warm and humid regions along the southern coast of Korea and on Jeju Island [2]. Despite its relatively restricted geographic distribution, considerable variation in plant growth and secondary metabolite accumulation was observed among the 17 collection sites. These differences may reflect the combined influences of regional climate, soil properties, local environmental conditions, and cultivation practices. Variation in plant age and developmental stage may also have contributed to differences in growth characteristics because tree age was not directly assessed in this study. Therefore, the objective of this study was not to rank collection sites according to plant growth performance, but to investigate associations among environmental conditions, growth characteristics, and the accumulation of major secondary metabolites under field conditions. The inclusion of both cultivated and naturally occurring populations allowed these relationships to be examined across a range of environmental conditions, although the contributions of individual factors could not be completely separated.

The predominance of rutin over chlorogenic acid (CGA) observed in most samples was consistent with previous reports on *D. trifidus* leaves. Youn et al. (2018) [21] reported consistently higher rutin than CGA concentrations in *D. morbifera* leaf extracts prepared from samples collected in different seasons using different extraction solvents. Similarly, Hwang et al. (2020) [22] reported higher rutin than CGA concentrations in leaf extracts prepared using different ethanol concentrations. Although absolute concentrations varied among studies, potentially because of differences in extraction procedures, plant age, and sampling season, the repeated observation of rutin predominance suggests that its relative abundance may be a characteristic feature of the phytochemical profiles examined under these conditions. A similar pattern was observed in the present study, with rutin generally accounting for a greater proportion of the combined CGA and rutin content despite substantial variation in total compound content among collection sites.

An exception was observed at sites 15–17, where CGA concentrations exceeded those of rutin and the total content of the two compounds was relatively low. These sites represented naturally occurring populations located at elevations of 308–535 m, in contrast to the predominantly cultivated populations examined in this study. This finding suggests that the relative accumulation of CGA and rutin may vary among *D. trifidus* populations, rather than consistently exhibiting rutin predominance. However, the factors underlying these differences remain unclear, as the observed phytochemical profiles may reflect variation in environmental conditions, plant age, or other population-specific characteristics. Further studies involving a broader range of natural and cultivated populations are needed to clarify the factors associated with variation in the relative accumulation of these compounds.

The multivariate analyses provided an exploratory overview of the relationships among plant growth, secondary metabolite accumulation, and environmental conditions. In the principal component analysis (PCA) based on 17 site-level observations, the first two principal components explained 51.43% of the total variance. CGA, rutin, and total compound content were oriented in similar directions, suggesting broadly consistent variation in their phytochemical profiles across the sampled sites. This pattern was also supported by the significant positive correlations among the major compound variables. Both CGA and rutin are derived from the phenylpropanoid biosynthetic pathway, which may contribute to their coordinated accumulation [23]. However, because total compound content was calculated from CGA and rutin, its close association with these individual compounds should not be interpreted as independent evidence of common metabolic regulation. Several environmental variables, including altitude (ALT), soil organic matter (OM), cation exchange capacity (CEC), and total nitrogen (TN), were oriented approximately opposite to the major compound vectors, indicating contrasting patterns in the PCA space. In addition, tree height (TH) was oriented toward the positive directions of both PC1 and PC2, whereas leaf length (LL) and dry weight of 10 leaves (10LDW) were oriented primarily toward the negative direction of PC2. These patterns illustrate variation in the multivariate relationships among compound accumulation, plant growth, and environmental conditions. Nevertheless, PCA represents an exploratory analysis, and the observed vector orientations should not be interpreted as evidence of statistically significant pairwise correlations or direct physiological relationships.

The site-level correlation analysis provided additional information on the relationships between growth characteristics and environmental conditions. TH was positively correlated with ALT, while the number of lobes (LobN) and leaf width (LW) was also positively correlated with ALT. Furthermore, 10LDW was positively correlated with available phosphorus (AP), suggesting an association between soil phosphorus availability and leaf dry biomass across the sampled sites (Figure 5 and Tables S6–S8). However, these relationships should not be interpreted as evidence that the corresponding environmental factors directly regulate plant growth. In contrast to the original individual-level analysis, the revised site-level analysis did not identify statistically significant correlations between rutin content and TH, OM, or CEC after FDR correction. Nevertheless, the negative correlation coefficients between rutin and these variables, together with their contrasting orientations in the PCA biplot, may be considered in the context of the growth–differentiation balance (GDB) hypothesis. This hypothesis proposes that resource availability may influence the balance between vegetative growth and secondary metabolism [24]. Although the observed patterns are not inconsistent with this framework, the absence of statistically significant associations after FDR correction provides insufficient evidence to support a growth–metabolite trade-off in the present study. Accordingly, the GDB hypothesis should be regarded only as a tentative interpretive framework rather than a mechanism demonstrated by the present results.

The relationships between environmental variables and the accumulation of rutin and CGA also require cautious interpretation. At the site level, rutin showed a positive correlation coefficient with annual maximum temperature (AMT) and negative correlation coefficients with ALT, OM, and CEC. Similarly, CGA showed a negative correlation coefficient with total precipitation (TP). However, none of these associations remained statistically significant after FDR correction. Previous studies have reported that flavonols such as rutin and quercetin can respond to elevated temperature or heat stress, potentially contributing to antioxidant defense and membrane protection [25,26]. Other studies have linked CGA accumulation with plant responses to drought or water-deficit conditions [27]. Although these findings provide possible biological explanations for the directions of the observed correlations, the present results do not provide sufficient statistical evidence to establish specific relationships between AMT and rutin or between TP and CGA. Further investigation is therefore required to determine whether these environmental factors are associated with consistent changes in compound accumulation in *D. trifidus*.

Both rutin and CGA are phenylpropanoid-derived compounds with antioxidant functions and may participate in plant responses to various environmental stresses. The significant positive correlation between CGA and rutin suggests that their accumulation varied in broadly similar directions among the sampled sites. However, this association does not necessarily indicate that the two compounds are regulated by identical physiological mechanisms. Their respective biosynthetic branches may respond differently to environmental conditions, developmental processes, or other factors not directly evaluated in this study. Although differences in their relative accumulation were observed among collection sites, the absence of statistically significant compound–environment correlations after FDR correction limits conclusions regarding specific environmental controls. Further physiological and molecular studies will be required to clarify whether differences in phenylpropanoid pathway regulation contribute to the observed variation in CGA and rutin accumulation.

Several limitations should be considered when interpreting these field-based results. First, this study was observational, and the identified associations cannot establish causal relationships between environmental conditions, plant growth, and secondary metabolite accumulation. Second, although 67 individual plant samples were collected, Pearson correlation analysis and PCA were performed using site-level observations (*n* = 17) to account for the hierarchical sampling structure and avoid pseudoreplication associated with repeated site-level climatic measurements. Consequently, the relatively small number of sampling sites, together with FDR correction for multiple comparisons, may have limited the statistical power to detect associations between compound contents and environmental variables. Several environmental variables were also intercorrelated, suggesting potential multicollinearity and shared environmental gradients. Because Pearson correlation analysis evaluates bivariate associations, the independent contributions of individual environmental factors to plant growth and major compound accumulation could not be determined, and the observed associations may partly reflect shared environmental gradients. Separate one-way ANOVAs were also performed for multiple growth and phytochemical variables. Although Tukey’s HSD test accounted for pairwise comparisons within each variable, the possibility of inflated Type I error across multiple response variables cannot be excluded. Third, plant age was not determined, although age-related variation may influence both plant growth and secondary metabolite accumulation. In particular, differences in tree height may partly reflect variation in plant age or developmental stage, making it difficult to distinguish age-related growth variation from environmental associations. Fourth, climatic variables were obtained from nearby meteorological stations rather than measured directly at each collection site and may not fully represent the microclimatic conditions experienced by individual plants. In addition, cultivation status may represent a potential confounding factor because the study included 14 cultivated sites and only three natural habitats. Differences in cultivation practices, including fertilization, irrigation, and pruning, as well as plant age and genetic background, may have contributed to the observed variation in plant growth and phytochemical composition. Furthermore, the three natural populations were located at relatively high elevations, making it difficult to distinguish the effects of cultivation status from those associated with altitude and other environmental conditions. Therefore, the distinct phytochemical profiles observed at these sites should be interpreted as population-level differences rather than being attributed to a specific environmental or cultivation-related factor. Finally, leaf samples were collected during a single sampling period in October. Although *D. trifidus* is an evergreen broad-leaved tree, leaf retention does not necessarily indicate that secondary metabolite concentrations remain constant throughout the year. Therefore, seasonal variation in CGA and rutin accumulation and the temporal consistency of their relationships with environmental conditions could not be evaluated. Some compound concentrations fell outside the validated calibration ranges; therefore, the corresponding quantitative results should be interpreted with caution.

Future studies should incorporate a larger number of geographically and environmentally diverse populations, together with age-controlled sampling, to better characterize variation in growth and secondary metabolite accumulation. Increasing the number of independent sampling sites would also improve the statistical power to evaluate environmental associations while accounting for site-level variation. Repeated sampling across multiple seasons and, ideally, multiple years would help characterize the temporal dynamics of CGA and rutin accumulation. Direct measurements of microclimatic conditions at individual sites would provide more precise estimates of the environments experienced by *D. trifidus*. Furthermore, controlled experiments involving temperature, water availability, and soil nutrient conditions could help determine whether the exploratory associations observed in this study reflect direct physiological responses. Measurements of physiological stress indicators and analyses of genes and enzymes involved in the phenylpropanoid pathway would also help clarify the mechanisms underlying CGA and rutin accumulation. Such approaches would strengthen the scientific basis for understanding environmental variation in *D. trifidus* and developing cultivation strategies aimed at producing leaves with desirable phytochemical profiles.

## 4. Materials and Methods

### 4.1. Sampling of Plant Material and Chemicals

**Figure 6:**
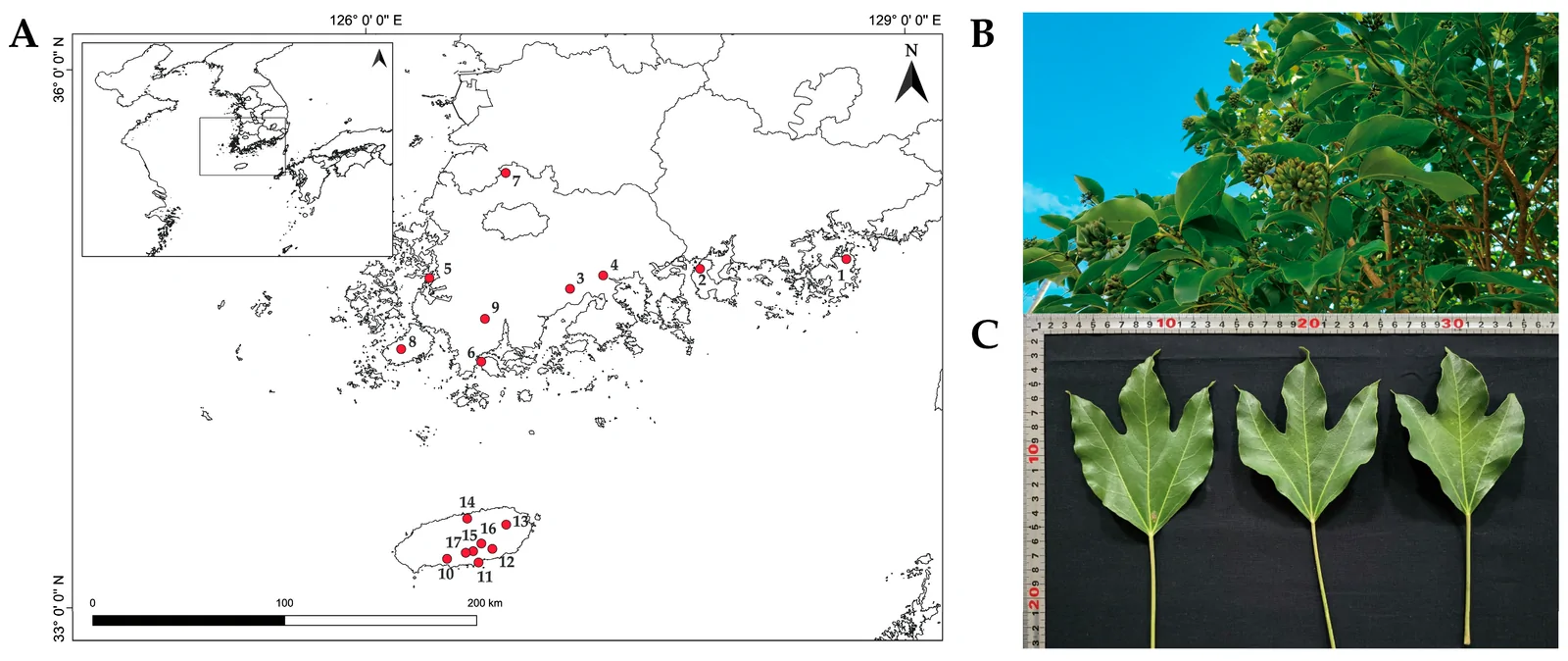
Geographical distribution and morphological characteristics of *Dendropanax trifidus*, illustrating the sampling locations across South Korea (**A**), the natural habitat in the field (**B**), and the detailed morphology of the collected leaves (**C**). The numbers in (**A**) indicate sampling site IDs, and the corresponding location information is provided in Table S9.

In October 2024, leaf and soil samples were collected from 17 sites in South Korea, comprising 14 cultivated sites and three natural habitats of *Dendropanax trifidus* (Figure 6 and Table S9). Four individual trees were sampled at each site, except at Site 16, where one sample was excluded because it was unsuitable for analysis. Consequently, a total of 67 individual trees were included in the study, representing biological replicates. Four two-year-old branches were collected from each sampled tree when the fruits were green, and the leaves attached to these branches were harvested without standardizing canopy position or orientation. Plant material was collected in excess of the amount required for growth and phytochemical analyses, and approximately three voucher specimens were prepared per sampling site using portions of the plant material collected from the sampled trees. No separate individual trees were designated specifically for voucher preparation. Voucher specimens were prepared and deposited in the herbarium of the Forest Medicinal Resources Research Center (FMRC), National Institute of Forest Science, Republic of Korea.

Eight growth-related traits were recorded for each population, including leaf length (LL), leaf width (LW), number of lobes (LobN), petiole length (PL), dry weight of 10 leaves (10LDW), moisture content (MC), tree height (TH), and root collar diameter (RCD). Four leaves per tree were used to measure LL, LW, LobN, and PL. LL, LW, and PL were measured using a digital caliper (model 500-182-30, Mitutoyo Co., Kawasaki, Japan). 10LDW was determined using an electronic balance (HS3200S, HANSUNG Instrument Co., Gwangmyeong, Republic of Korea), and moisture content (MC) was calculated from fresh and dry weights. TH was measured using a clinometer (SUUNTO, Vantaa, Finland), and RCD was measured using a diameter tape.

HPLC-grade acetonitrile, methanol, and water were obtained from J.T. Baker (Avantor Inc., Radnor, PA, USA). Chlorogenic acid (≥98%, CAS No. 327-97-9) and rutin (≥98%, CAS No. 153-18-4) were purchased from ChemFaces (Wuhan, Hubei, China) and used as reference standards.

### 4.2. Sample and Standard Preparation

Leaf samples were washed and lyophilized (−80 °C, 5 days, LP20, ilShinBioBase Co., Ltd., Dongducheon, Republic of Korea). The lyophilized samples were weighed for calculated MC and subsequently pulverized using a KSP-35 pulverizer (Korea Medi Co., Ltd., Daegu, Republic of Korea). For extraction, 1 g of powdered sample was transferred to a 50 mL tube and mixed with 20 mL of methanol. The suspension was vortex-mixed and sonicated for 1 h at 30 °C using a water bath sonicator (350 W, 40 kHz; JAC-5020, KODO Technical Research Co., Ltd., Hwaseong, Republic of Korea). Following extraction, the samples were centrifuged at 1763× *g* for 15 min (UM-1248, Labogene, Seoul, Republic of Korea). The resulting supernatants were passed through qualitative filter paper (ADVENTEC^®^ No. 2, Toyo Roshi Kaisha, Ltd., Tokyo, Japan) and stored at 4 °C until analysis. Immediately before UPLC analysis, the extracts were further filtered through 0.2 μm PTFE syringe filters (Cat. No. 6784-1302, Whatman Co., Maidstone, UK). Standard stock solutions of chlorogenic acid (CGA) and rutin were prepared in methanol and serially diluted to obtain the required calibration concentrations.

### 4.3. UPLC Analysis and Validation

Quantitative analysis of the major compounds was performed using a Waters Alliance UPLC-PDA system (Waters Co., Milford, MA, USA). Chromatographic separation was achieved using a TSS H3 column (2.1 × 100 mm, 1.7 μm, 130 Å; Waters Co., Milford, MA, USA) maintained at 30 °C. The mobile phase consisted of water containing 0.1% formic acid (A) and acetonitrile containing 0.1% formic acid (B). The gradient program was as follows: 10% B from 0 to 2 min, 10% B from 2 to 7 min, 15% B from 7 to 14 min, 20% B from 14 to 16 min, and 100% B from 16 to 16.1 min. The injection volume was 1 μL, the flow rate was 0.2 mL/min, and ultraviolet detection was performed at 254 nm. Chromatographic data were acquired and processed using Empower 3 software (Waters Co., Milford, MA, USA). Each sample was extracted once, and the resulting extract was injected three times into the UPLC-PDA system. The mean of the three injections was used for subsequent analyses. CGA and rutin were identified by comparing their retention times and UV absorption spectra with those of authentic reference standards during UPLC-PDA method establishment. The contents of CGA and rutin were expressed as milligrams per gram of dry leaf weight (mg/g DW). Compound concentrations determined by UPLC-PDA were converted to dry-weight-based contents using the extraction volume, dry sample weight, and dilution factor, according to the following equation:

(1) $$\mathrm{Compound}\;\mathrm{content}\left(\mathrm{mg}/g\;\mathrm{DW}\right)=\frac{\left(C\times V\times \mathrm{DF}\right)}{W}$$

where *C* is the measured concentration (mg/mL), *V* is the final extraction volume (mL), *DF* is the dilution factor, and *W* is the dry weight of the extracted leaf sample (g).

The analytical method was validated in accordance with the International Council for Harmonisation of Technical Requirements for Pharmaceuticals for Human Use (ICH) guidelines [28]. Selected analytical performance characteristics, including linearity, limits of detection (LOD) and quantification (LOQ), and intra- and inter-day precision, were evaluated with reference to the ICH Q2(R2) guideline. CGA and rutin peaks in the leaf extracts were identified by comparing their retention times with those of authentic reference standards. Linearity was evaluated using calibration curves prepared at six concentration levels (25–800 μg/mL) for CGA and seven concentration levels (5–800 μg/mL) for rutin. The coefficients of determination (R^2^) were used to assess the linearity of the calibration curves. The limits of detection (LOD) and quantification (LOQ) were calculated as 3.3σ/S and 10σ/S, respectively, where σ represents the standard deviation of the response and S represents the slope of the calibration curve. Method precision was evaluated using relative standard deviation (RSD, %) at three concentration levels. Each concentration was analyzed in triplicate within a single day for intra-day precision and on different days for inter-day precision.

### 4.4. Environmental Data

Soil samples were collected separately from each sampled tree at a depth of 20 cm after removal of the surface layer. The collected soils were air-dried under shade and homogenized after passing through a 2 mm sieve. Soil texture was classified according to the USDA soil classification system. Soil physicochemical properties were analyzed following the standard analytical procedures of the Rural Development Administration of Korea [29]. Soil pH and electrical conductivity (EC) were measured using a 1:5 (*w*/*v*) soil-to-water suspension prepared by mixing 10 g of air-dried soil with 50 mL of distilled water. Soil organic matter (OM) was determined using the Tyurin method. Available phosphorus (AP), expressed as P_2_O_5_, was quantified colorimetrically using the molybdenum blue method with 1-amino-2-naphthol-4-sulfonic acid. Exchangeable potassium (K^+^), calcium (Ca^2+^), magnesium (Mg^2+^), and sodium (Na^+^) were extracted using 1 N ammonium acetate (NH_4_OAc) adjusted to pH 7.0, and the concentrations in the extracts were determined by inductively coupled plasma optical emission spectrometry (ICP-OES). Cation exchange capacity (CEC) was determined by ammonium acetate saturation followed by Kjeldahl distillation. Briefly, the exchange sites of the soil were saturated with NH_4_^+^ using ammonium acetate, and the amount of NH_4_^+^ retained by the soil was quantified by Kjeldahl distillation to estimate the CEC. Total nitrogen (TN) was determined using the Kjeldahl sulfuric acid digestion–distillation method. Each soil sample was analyzed once, and the results from the independent samples were used to calculate the site-level means ± standard errors (SE).

Meteorological data, including annual average temperature (AAT), annual average maximum temperature (AAMT), annual average minimum temperature (AAmT), annual maximum temperature (AMT), annual minimum temperature (AmT), total precipitation (TP), relative humidity (RH), and sunshine hours (SSH), were obtained from 11 Automated Synoptic Observing System (ASOS) stations through the open data platform of the Korea Meteorological Administration (KMA; data.kma.go.kr). Annual meteorological data for 2024, the year of sample collection, were used, and each sampling site was matched to the nearest meteorological station based on geographical distance. The meteorological data were accessed on 2 September 2025.

### 4.5. Statistical Analysis

Statistical analyses were conducted using IBM SPSS Statistics version 26.0 (IBM Corp., Armonk, NY, USA) and R version 4.2.3 (R Foundation for Statistical Computing, Vienna, Austria). Data are presented as mean ± standard error (SE), and statistical significance was considered at *p* < 0.05. Differences in growth characteristics and major compound contents among collection sites were evaluated using one-way analysis of variance (ANOVA) for each response variable, followed by Tukey’s honestly significant difference (HSD) test for pairwise comparisons among collection sites. For Pearson correlation analysis and principal component analysis (PCA), site-level mean values were used to account for the hierarchical sampling structure and avoid pseudoreplication. Phytochemical contents, growth characteristics, and soil physicochemical properties measured from individual trees were averaged within each sampling site, whereas meteorological variables and altitude were retained at the site level. Consequently, 17 site-level observations (*n* = 17) were used for both analyses. PCA was performed to investigate the multivariate relationships among major compound contents, growth characteristics, soil physicochemical properties, and meteorological variables. Prior to analysis, the variables were standardized to a mean of zero and a standard deviation of one. PCA results were summarized and visualized using the factoextra (ver. 1.0.7) and ggplot2 (ver. 4.0.2) packages in R. The first two principal components (PC1 and PC2) were selected for biplot visualization because they accounted for the largest proportions of total variance. Eigenvalues, explained variance, and cumulative explained variance for the principal components were provided in Table S5. PCA was used as an exploratory method to visualize multivariate patterns rather than to establish statistically significant relationships among individual variables. Pearson’s correlation coefficients were calculated to evaluate the relationships among major compound contents, growth characteristics, and environmental variables. To account for multiple testing, *p*-values for all unique pairwise correlations among the 33 variables were adjusted collectively using the Benjamini–Hochberg false discovery rate (FDR) procedure. The resulting correlation coefficients and adjusted *p*-values were presented in separate supplementary tables according to variable categories for clarity. Correlation matrices were generated and visualized using the ggcorrplot2 (ver. 0.1.2) and qgraph (ver. 1.9.8) packages in R version 4.2.3 within the RStudio environment (Posit, Boston, MA, USA).

## 5. Conclusions

This study demonstrated substantial site-to-site variation in the growth characteristics and major phenolic compound contents of *Dendropanax trifidus* across cultivated and natural populations in southern Korea and Jeju Island. Rutin generally predominated over chlorogenic acid (CGA), whereas the relative accumulation of these compounds differed at the high-elevation natural sites, where total compound contents were also comparatively low. Site-level correlation analysis identified significant positive associations between altitude and tree height, altitude and leaf lobe number, and soil available phosphorus and leaf dry weight. In contrast, no significant correlations between compound contents and the measured environmental or growth variables remained after FDR correction. These findings highlight variation in the growth and phytochemical characteristics of *D. trifidus* across collection sites, while indicating that the environmental factors associated with compound accumulation remain uncertain. Although the observational design and limited number of sampling sites restrict the interpretation of these relationships, the present findings provide preliminary information for future investigations into suitable cultivation conditions and targeted compound production. Further studies involving additional sampling sites and controlled environmental conditions are needed to clarify the factors influencing CGA and rutin accumulation.

## Figures and Tables

**Table 1:** Growth characteristics of *Dendropanax trifidus* in 17 different sites.

Collection Sites	LL	LW	LobN	PL	10LDW	MC	TH	RCD
	(mm)	(mm)		(mm)	(g)	(%)	(m)	(cm)
1	127.52 ± 9.78 ^ab^	102.66 ± 2.20 ^bc^	0.92 ± 0.46 ^abc^	131.45 ± 11.44 ^a^	5.15 ± 0.70 ^d^	29.99 ± 0.49 ^d^	3.50 ± 0.72 ^efg^	7.30 ± 0.84 ^bcd^
2	115.68 ± 7.22 ^b^	81.21 ± 8.56 ^bc^	0.33 ± 0.33 ^bc^	74.35 ± 9.50 ^a^	4.65 ± 0.52 ^d^	34.40 ± 0.84 ^cd^	6.20 ± 0.78 ^cdef^	10.75 ± 1.83 ^abc^
3	120.63 ± 3.55 ^b^	82.72 ± 7.77 ^bc^	0.67 ± 0.38 ^abc^	91.20 ± 18.59 ^a^	5.64 ± 0.68 ^cd^	33.69 ± 2.13 ^cd^	6.38 ± 0.56 ^cde^	12.10 ± 2.62 ^ab^
4	126.64 ± 9.19 ^ab^	93.00 ± 12.90 ^bc^	0.50 ± 0.50 ^abc^	100.40 ± 15.74 ^a^	7.75 ± 1.16 ^bcd^	36.90 ± 1.11 ^bcd^	2.88 ± 0.32 ^fg^	10.20 ± 0.62 ^abcd^
5	126.34 ± 3.86 ^b^	100.58 ± 8.50 ^bc^	0.75 ± 0.44 ^abc^	92.01 ± 5.25 ^a^	6.68 ± 0.32 ^bcd^	34.67 ± 1.15 ^bcd^	4.30 ± 0.00 ^cdefg^	12.88 ± 1.21 ^ab^
6	160.96 ± 5.17 ^a^	127.41 ± 12.06 ^ab^	0.75 ± 0.44 ^abc^	133.52 ± 10.52 ^a^	15.33 ± 1.76 ^a^	32.98 ± 1.60 ^abc^	2.40 ± 0.20 ^g^	10.05 ± 0.73 ^abcd^
7	116.04 ± 5.36 ^b^	72.80 ± 3.82 ^c^	0.00 ± 0.00 ^c^	71.71 ± 9.06 ^a^	4.76 ± 0.35 ^d^	34.23 ± 1.17 ^cd^	4.65 ± 0.53 ^cdefg^	15.08 ± 0.58 ^a^
8	123.35 ± 2.26 ^b^	92.77 ± 5.38 ^bc^	0.67 ± 0.38 ^abc^	100.59 ± 9.12 ^a^	6.19 ± 0.32 ^bcd^	34.48 ± 0.15 ^cd^	3.30 ± 0.13 ^efg^	11.78 ± 0.71 ^ab^
9	126.69 ± 4.67 ^ab^	83.82 ± 4.13 ^bc^	0.00 ± 0.00 ^c^	94.88 ± 5.46 ^a^	6.23 ± 1.05 ^bcd^	36.11 ± 1.47 ^cd^	3.60 ± 0.60 ^efg^	13.85 ± 0.37 ^a^
10	138.71 ± 10.93 ^ab^	89.83 ± 7.69 ^bc^	0.17 ± 0.17 ^bc^	93.16 ± 13.16 ^a^	8.30 ± 1.47 ^bcd^	31.26 ± 0.49 ^abcd^	7.75 ± 1.11 ^bc^	9.45 ± 1.08 ^abcd^
11	138.92 ± 6.73 ^ab^	96.77 ± 8.42 ^bc^	0.25 ± 0.25 ^bc^	89.07 ± 6.90 ^a^	11.62 ± 0.84 ^ab^	34.80 ± 0.58 ^ab^	3.88 ± 0.43 ^defg^	15.20 ± 1.39 ^a^
12	124.55 ± 9.03 ^ab^	93.51 ± 12.80 ^bc^	0.42 ± 0.32 ^abc^	80.01 ± 12.50 ^a^	5.54 ± 1.23 ^cd^	27.75 ± 4.47 ^cd^	10.00 ± 0.41 ^ab^	15.75 ± 0.88 ^a^
13	140.40 ± 8.18 ^ab^	85.13 ± 2.22 ^bc^	0.00 ± 0.00 ^c^	85.46 ± 10.64 ^a^	8.73 ± 0.50 ^bcd^	32.26 ± 2.34 ^abcd^	3.25 ± 0.15 ^efg^	4.15 ± 0.15 ^d^
14	133.17 ± 7.71 ^ab^	89.00 ± 6.68 ^bc^	0.31 ± 0.19 ^bc^	80.71 ± 5.47 ^a^	11.85 ± 1.80 ^abc^	34.38 ± 0.81 ^a^	3.13 ± 0.13 ^efg^	4.45 ± 0.27 ^cd^
15	126.74 ± 8.91 ^ab^	126.38 ± 5.08 ^ab^	1.67 ± 0.33 ^ab^	90.87 ± 6.44 ^a^	5.76 ± 0.64 ^bcd^	32.93 ± 1.36 ^cd^	10.67 ± 2.03 ^a^	10.60 ± 2.17 ^ab^
16	145.59 ± 7.40 ^ab^	154.81 ± 12.17 ^a^	2.00 ± 0.00 ^a^	111.31 ± 4.34 ^a^	7.52 ± 1.74 ^bcd^	34.24 ± 2.47 ^cd^	11.67 ± 1.21 ^a^	12.37 ± 0.90 ^ab^
17	147.78 ± 8.41 ^ab^	148.00 ± 8.94 ^a^	2.00 ± 0.00 ^a^	119.11 ± 16.28 ^a^	6.07 ± 1.11 ^bcd^	33.54 ± 2.76 ^d^	7.13 ± 0.43 ^bcd^	4.95 ± 0.73 ^cd^

LL, leaf length; LW, leaf width; LobN, number of lobes; PL, petiole length; 10LDW, 10 leaves dry weight; MC, moisture contents; TH, tree height; RCD, root collar diameter. Values are presented as mean ± standard error (SE) based on four biological replicates per site, except at Site 16, where three biological replicates were analyzed. Tukey’s Honestly Significant Difference (HSD) multiple comparison tests showed that there were statistically significant differences in means between groups with different letters (*p* < 0.05).

**Table 2:** Linear regression, LOD, and LOQ of two major compounds in *D. trifidus* leaves.

Compound	Regression Equation	Correlation Coefficient (*r*^2^)	Range (µg/mL)	LOD (µg/mL)	LOQ (µg/mL)
Chlorogenic acid	Y = 2233.1X + 469.77	1.000	25–800	0.49	1.50
Rutin	Y = 3716.5X − 2047.70	1.000	5–800	0.03	0.10

**Table 3:** Intra- and inter-day precision of two major compounds in *D. trifidus* leaves.

Compound	Concentration (µg/mL)	Intra-Day ^a^		Inter-Day ^b^	
		Concentration Found (µg/mL)	RSD (%)	Concentration Found (µg/mL)	RSD (%)
Chlorogenic acid	25	24.41	0.24	24.38	0.30
	50	49.19	0.21	49.15	0.20
	100	101.00	0.15	101.09	0.05
Rutin	25	25.62	0.20	25.64	0.17
	50	49.54	0.26	49.47	0.26
	100	98.70	0.20	98.73	0.24

^a^ Sample was analyzed 3 times in 1 day, *n* = 3; ^b^ Sample was analyzed each day on 3 consecutive days, *n* = 3; RSD, relative standard deviation.

## Data Availability

The data supporting the findings of this study are included in the article and its Supplementary Materials. Additional data, including precise sampling coordinates and individual-level raw data, are available from the corresponding author upon reasonable request.

## Supplementary Materials

The following supporting information can be downloaded at: https://www.mdpi.com/article/10.3390/plants15213415/s1, Table S1: Soil physicochemical data of 17 different sites of *Dendropanax trifidus*; Table S2. Soil particle-size distribution and texture classification across the 17 collection sites of *Dendropanax trifidus*; Table S3: Meteorological data for 17 different sites of *Dendropanax trifidus*; Table S4: Concentrations of major compounds in *Dendropanax trifidus* leaves; Table S5: Eigenvalues, explained variance, and cumulative explained variance of principal components obtained from PCA using site-level mean values (*n* = 17); Table S6: Pearson’s correlation coefficients between the growth characteristics of *Dendropanax trifidus* and soil physicochemical properties; Table S7: Pearson’s correlation coefficient between the growth characteristics of *Dendropanax trifidus* leaves and meteorological factors; Table S8: Pearson’s correlation coefficient between major compounds and the growth characteristics of *Dendropanax trifidus* leaves; Table S9: Geographic information about the collection sites where leaves of *Dendropanax trifidus* were collected in South Korea, including the corresponding meteorological stations and distances.

## Abbreviations

The following abbreviations are used in this manuscript:

AAMT	Annual average maximum temperature
AAmT	Annual average minimum temperature
AAT	Annual average temperature
ALT	Altitude
AMT	Annual maximum temperature
AmT	Annual minimum temperature
AP	Available phosphate
CEC	Cation exchange capacity
CGA	Chlorogenic acid
EC	Electrical conductivity
LL	Leaf length
LobN	Number of lobes
LOD	Limit of detection
LOQ	Limit of quantification
LW	Leaf width
MC	Moisture contents
OM	Organic matter
PL	Petiole length
RCD	Root collar diameter
RH	Relative humidity
RSD	Relative standard deviation
SSH	Sunshine hours
TH	Tree height
TN	Total nitrogen
TP	Total precipitation
10LDW	10 leaves dry weight
