# Supplementary material for: Environmental Factors Associated with Growth and Major Compound Accumulation in Dendropanax trifidus
Source: Plants (Basel). 2026 Nov 3;15(21):3415. doi: 10.3390/plants15213415 (PMC13766133; doi:10.3390/plants15213415)
Supplement: Supplementary file 1 [file plants-15-03415-s001.zip › plants-4604484-supplementary.pdf]

SUPPLEMENTARY DATA

Environmental Factors Associated with Growth and Major Compound Accumulation in *Dendropanax trifidus*

Dong Hwan Lee [1], Jun Hyuk Jang [1], Ji Ah Kim [2,*]

[1] Forest Medicinal Resources Research Center, National Institute of Forest Science, Yeongju-si 36040, Republic of Korea; wnseldu123@korea.kr (J.H.J.)

[2] Department of Forest Bioresources, National Institute of Forest Science, 39 Onjeong-Ro, Gwonseon-Gu, Suwon 16631, Republic of Korea

* Correspondence: jiahkim@korea.kr

Table S1. Soil physicochemical data of 17 different sites of *Dendropanax trifidus*.

Collection sites	pH	EC	OM	TN	AP	K^+	Ca^{2+}	Mg^{2+}	Na^+	CEC
		(dS/m)	(%)		(mg/kg)	($cmol^+$/kg)				
1	5.43±0.29^{abc}	0.85±0.07^{c}	4.02±0.70^{c}	0.18±0.03^{f}	292.54±100.03^{cde}	0.57±0.14^{ab}	12.78±5.26^{a}	6.01±2.61^{a}	0.14±0.03^{b}	17.12±2.61^{d}
2	4.69±0.09^{bcde}	0.87±0.02^{c}	2.99±0.19^{c}	0.17±0.02^{f}	401.30±60.54^{bcde}	0.56±0.12^{ab}	5.48±0.60^{ab}	3.12±0.67^{ab}	0.06±0.01^{b}	16.17±0.77^{d}
3	4.51±0.16^{de}	0.84±0.04^{c}	3.23±0.17^{c}	0.15±0.02^{f}	185.37±28.32^{cde}	0.36±0.07^{abc}	2.40±0.66^{c}	0.55±0.07^{b}	0.07±0.01^{b}	13.25±0.27^{d}
4	5.29±0.12^{abcd}	1.16±0.14^{c}	3.29±0.60^{c}	0.16±0.03^{f}	334.13±56.82^{bcde}	0.41±0.07^{abc}	7.18±0.58^{ab}	1.44±0.26^{b}	0.08±0.02^{b}	14.57±1.75^{d}
5	6.07±0.26^{a}	1.20±0.08^{c}	3.66±0.24^{c}	0.18±0.02^{f}	513.88±34.91^{abcd}	0.65±0.18^{a}	8.19±0.49^{ab}	1.73±0.17^{b}	0.08±0.01^{b}	14.77±1.21^{d}
6	5.49±0.06^{ab}	0.78±0.10^{c}	2.14±0.42^{c}	0.12±0.03^{f}	737.56±114.71^{ab}	0.26±0.01^{abc}	5.95±0.50^{ab}	1.18±0.16^{b}	0.10±0.02^{b}	12.68±0.49^{d}
7	5.24±0.40^{abcd}	0.96±0.06^{c}	4.08±0.32^{c}	0.19±0.01^{f}	167.79±25.19^{cde}	0.23±0.02^{bc}	5.61±2.21^{ab}	0.72±0.11^{b}	0.11±0.04^{b}	15.54±0.86^{d}
8	5.28±0.20^{abcd}	0.82±0.01^{c}	2.19±0.11^{c}	0.12±0.01^{f}	168.02±19.20^{cde}	0.28±0.01^{abc}	8.28±0.83^{ab}	3.42±0.24^{ab}	0.38±0.05^{a}	14.21±0.97^{d}
9	4.60±0.04^{cde}	0.89±0.06^{c}	3.84±0.22^{c}	0.18±0.02^{f}	130.06±33.57^{cde}	0.26±0.04^{abc}	1.12±0.31^{c}	0.50±0.07^{b}	0.07±0.01^{b}	13.44±0.37^{d}
10	4.81±0.06^{bcde}	0.95±0.03^{c}	23.59±0.47^{b}	0.52±0.02^{cde}	107.86±5.60^{de}	0.26±0.04^{abc}	0.92±0.12^{c}	0.49±0.07^{b}	0.13±0.02^{b}	35.97±0.44^{c}
11	4.62±0.05^{cde}	1.21±0.06^{c}	25.00±0.43^{b}	0.65±0.05^{bcd}	552.57±201.12^{abc}	0.49±0.10^{abc}	1.49±0.23^{c}	0.69±0.13^{b}	0.11±0.01^{b}	39.17±2.45^{bc}
12	4.55±0.08^{de}	1.03±0.10^{c}	23.14±0.34^{b}	0.55±0.02^{cd}	125.31±8.56^{cde}	0.18±0.02^{bc}	0.73±0.33^{c}	0.41±0.15^{b}	0.20±0.09^{b}	39.31±1.75^{bc}
13	4.66±0.05^{bcde}	2.45±0.39^{a}	36.33±2.16^{a}	0.92±0.13^{ab}	123.68±19.62^{cde}	0.36±0.07^{abc}	3.28±0.88^{c}	1.44±0.26^{b}	0.20±0.03^{b}	49.23±2.10^{ab}
14	5.00±0.18^{bcde}	1.37±0.13^{bc}	4.15±0.64^{c}	0.23±0.05^{ef}	919.16±199.44^{a}	0.53±0.12^{ab}	5.54±1.89^{ab}	1.98±0.73^{b}	0.12±0.03^{b}	16.13±1.98^{d}
15	4.58±0.07^{cde}	1.33±0.07^{c}	26.21±2.43^{b}	0.50±0.03^{de}	55.08±11.49^{e}	0.10±0.02^{c}	0.51±0.08^{c}	0.40±0.03^{b}	0.10±0.02^{b}	40.13±4.25^{bc}
16	4.26±0.03^{e}	2.81±0.20^{a}	39.61±0.96^{a}	1.10±0.06^{a}	235.10±29.07^{cde}	0.34±0.04^{abc}	0.72±0.17^{c}	0.61±0.04^{b}	0.16±0.01^{b}	58.02±4.78^{a}
17	4.49±0.17^{de}	2.35±0.62^{ab}	35.26±4.42^{a}	0.80±0.19^{abc}	95.07±18.27^{de}	0.22±0.05^{bc}	0.71±0.33^{c}	0.52±0.09^{b}	0.21±0.06^{b}	50.16±6.33^{ab}

EC, electrical conductivity; OM, organic matter; TN, total nitrogen; AP, available phosphate; CEC, cation exchange capacity. Values are presented as mean ± standard error (SE) based on four biological replicates per site, except at Site 16, where three biological replicates were analyzed. Tukey's Honestly Significant Difference (HSD) multiple comparison tests showed that there were statistically significant differences in means between groups with different letters ($p < 0.05$).

Table S2. Soil particle-size distribution and texture classification across the 17 collection sites of *Dendropanax trifidus*.

Collection sites	Sand	Silt	Clay	Soil texture
		(%)		
1	45.31±1.59[a]	22.66±1.10[d]	32.03±1.57[a]	Clay loam
2	36.04±0.63[de]	34.12±0.45[a]	29.85±0.67[a]	Clay loam
3	43.61±0.71[abc]	25.47±0.56[cd]	30.92±1.25[a]	Clay loam
4	41.71±0.64[abc]	25.40±0.56[cd]	32.89±0.41[a]	Clay loam
5	41.65±0.78[abc]	25.41±0.47[cd]	32.94±0.48[a]	Clay loam
6	43.16±0.97[abc]	25.90±0.55[cd]	30.95±0.85[a]	Clay loam
7	42.12±0.70[abc]	26.67±0.45[cd]	31.22±1.15[a]	Clay loam
8	40.24±0.97[cd]	29.60±0.81[abc]	30.16±0.94[a]	Clay loam
9	42.24±0.48[abc]	25.89±1.09[cd]	31.87±0.68[a]	Clay loam
10	44.64±0.48[ab]	25.15±0.65[cd]	30.21±0.77[a]	Clay loam
11	35.13±0.55[e]	32.16±0.49[ab]	32.71±0.57[a]	Clay loam
12	36.51±0.28[de]	33.78±0.79[a]	29.71±0.57[a]	Clay loam
13	36.16±0.69[de]	32.93±1.08[a]	30.91±1.15[a]	Clay loam
14	42.56±0.82[abc]	25.85±0.69[cd]	31.59±0.37[a]	Clay loam
15	43.14±0.71[abc]	25.98±0.40[cd]	30.89±0.65[a]	Clay loam
16	35.23±0.78[e]	33.10±0.56[a]	31.68±1.14[a]	Clay loam
17	40.38±1.35[bcd]	27.63±2.53[bc]	31.99±1.55[a]	Clay loam

Values are presented as mean ± standard error (SE) based on four biological replicates per site, except at Site 16, where three biological replicates were analyzed. Tukey's Honestly Significant Difference (HSD) multiple comparison tests showed that there were statistically significant differences in means between groups with different letters ($p < 0.05$).

Table S3. Meteorological data for 17 different sites of *Dendropanax trifidus*.

Collection sites	AAT	AAMT	AAmT	AMT	AmT	TP	RH	SSH	ALT
			(°C)			(mm)	(%)	(h)	(m)
1	16.0	20.3	12.2	34.9	-6.0	2213.4	72.1	2038.1	67.8
2	16.0	20.5	12.2	35.7	-5.6	2375.1	71.9	2190.7	115.2
3	15.8	21.0	11.2	36.3	-5.5	1928.2	78.0	2254.8	74.0
4	15.8	21.0	11.2	36.3	-5.5	1928.2	78.0	2254.8	30.2
5	15.7	19.5	12.6	34.9	-4.9	1285.2	77.3	2127.6	33.7
6	16.5	21.5	12.6	37.0	-3.3	1963.7	74.5	2140.7	25.0
7	15.0	20.5	10.3	36.2	-8.4	1136.1	75.8	1887.9	197.8
8	15.5	20.0	11.4	34.7	-4.8	1719.3	78.8	1984.1	23.0
9	15.6	20.7	10.9	35.0	-6.2	1504.0	79.3	2258.8	91.8
10	18.3	21.7	15.5	34.7	-1.8	2247.3	71.8	2046.0	239.0
11	18.3	21.7	15.5	34.7	-1.8	2247.3	71.8	2046.0	10.0
12	18.3	21.7	15.5	34.7	-1.8	2247.3	71.8	2046.0	183.9
13	17.4	20.9	14.1	33.7	-2.1	2455.3	77.2	1849.8	214.0
14	18.2	21.4	15.4	35.5	-1.0	1597.7	67.5	1754.8	104.2
15	18.3	21.7	15.5	34.7	-1.8	2247.3	71.8	2046.0	308.0
16	18.3	21.7	15.5	34.7	-1.8	2247.3	71.8	2046.0	535.4
17	18.3	21.7	15.5	34.7	-1.8	2247.3	71.8	2046.0	534.7

AAT, annual average temperature; AAMT, annual average maximum temperature; AAmT, annual average minimum temperature; AMT, annual maximum temperature; AmT, annual minimum temperature; TP, total precipitation; RH, relative humidity; SSH, sum of sunshine hour; ALT, altitude.

Table S4. Concentrations of major compounds in *Dendropanax trifidus* leaves.

Sample No.	Collection Sites	Tree No.	Chlorogenic acid	Rutin	Total
				(mg/g DW)	
1	1	1	0.096	0.344	0.441
2		2	0.062	0.083	0.145
3		3	0.097	0.135	0.232
4		4	0.047	0.227	0.274
5	2	1	0.116	0.219	0.335
6		2	0.126	0.164	0.291
7		3	0.111	0.274	0.385
8		4	0.177	0.362	0.539
9	3	1	9.023	21.706	30.729
10		2	4.889	11.674	16.563
11		3	9.580	23.055	32.635
12		4	3.927	2.907	6.834
13	4	1	3.508	14.204	17.712
14		2	2.227	5.244	7.472
15		3	4.436	8.253	12.688
16		4	4.879	12.655	17.534
17	5	1	7.863	17.559	25.422
18		2	6.854	13.510	20.364
19		3	5.694	12.346	18.040
20		4	8.255	11.890	20.145
21	6	1	3.255	22.098	25.353
22		2	4.528	16.026	20.554
23		3	4.772	10.480	15.252
24		4	7.492	28.040	35.532
25	7	1	4.137	6.179	10.316
26		2	3.166	3.019	6.185
27		3	3.188	4.707	7.895
28		4	3.932	8.997	12.929
29	8	1	3.151	7.288	10.439
30		2	4.682	8.974	13.656
31		3	4.448	10.947	15.395
32		4	6.232	12.313	18.545
33	9	1	4.346	7.722	12.068
34		2	4.919	9.093	14.012
35		3	4.673	9.201	13.874
36		4	5.953	6.243	12.196
37	10	1	4.865	7.294	12.159
38		2	4.934	8.906	13.840
39		3	2.642	4.100	6.743
40		4	3.460	6.656	10.115

41	11	1	2.933	5.042	7.975
42		2	3.886	7.408	11.294
43		3	9.503	13.441	22.944
44		4	6.176	10.578	16.754
45	12	1	3.729	5.027	8.756
46		2	3.325	2.948	6.273
47		3	2.790	1.984	4.774
48		4	0.895	0.120	1.014
49	13	1	1.244	0.833	2.078
50		2	1.344	0.464	1.808
51		3	1.328	0.929	2.256
52		4	0.569	0.623	1.192
53	14	1	3.076	8.774	11.850
54		2	3.727	12.767	16.493
55		3	1.681	11.553	13.234
56		4	2.345	9.355	11.700
57	15	1	2.122	3.032	5.154
58		2	0.544	0.237	0.781
59		3	0.593	0.486	1.078
60		4	0.647	0.137	0.784
61	16	1	1.206	0.257	1.464
62		2	1.433	0.812	2.245
63		3	0.671	0.775	1.445
64	17	1	0.834	0.381	1.216
65		2	0.499	0.145	0.645
66		3	2.116	0.331	2.447
67		4	2.548	0.260	2.808

Table S5. Eigenvalues, explained variance, and cumulative explained variance of principal components obtained from PCA using site-level mean values (n = 17).

Principal component	Eigenvalue	Explained variance (%)	Cumulative variance (%)
PC1	12.500	37.88	37.88
PC2	4.474	13.56	51.43
PC3	3.653	11.07	62.50
PC4	2.883	8.74	71.24
PC5	2.255	6.83	78.07
PC6	1.789	5.42	83.49
PC7	1.481	4.49	87.98
PC8	1.141	3.46	91.44
PC9	1.024	3.10	94.54
PC10	0.591	1.79	96.33
PC11	0.446	1.35	97.68
PC12	0.251	0.76	98.44
PC13	0.209	0.63	99.07
PC14	0.130	0.39	99.46
PC15	0.113	0.34	99.81
PC16	0.063	0.19	100.00

PCA was performed using 33 standardized variables from 17 sampling sites. All 16 principal components with non-zero eigenvalues are presented. PC1 and PC2 were selected for exploratory biplot visualization.

Table S6. Pearson's correlation coefficients between the growth characteristics of *Dendropanax trifidus* and soil physicochemical properties.

Soil Properties	Correlation coefficient (*r*)[a]							
	Growth traits							
	LL	LW	LobN	PL	10LDW	MC	TH	RCD
pH	-0.092 (0.839)	-0.209 (0.607)	-0.220 (0.597)	0.211 (0.607)	0.157 (0.704)	0.122 (0.778)	-0.609 (0.074)	-0.002 (0.994)
EC	0.475 (0.190)	0.599 (0.084)	0.527 (0.135)	0.123 (0.777)	0.043 (0.925)	0.044 (0.925)	0.372 (0.338)	-0.410 (0.293)
OM	0.476 (0.190)	0.552 (0.112)	0.452 (0.225)	0.041 (0.929)	0.018 (0.968)	-0.297 (0.449)	0.613 (0.071)	-0.195 (0.638)
TN	0.489 (0.174)	0.543 (0.117)	0.426 (0.264)	0.043 (0.925)	0.054 (0.922)	-0.230 (0.575)	0.562 (0.107)	-0.188 (0.650)
AP	0.277 (0.483)	-0.028 (0.949)	-0.179 (0.669)	0.085 (0.854)	**0.724*** **(0.016)**	0.250 (0.541)	-0.490 (0.174)	-0.162 (0.699)
K^+	-0.168 (0.687)	-0.255 (0.528)	-0.214 (0.606)	-0.026 (0.949)	0.098 (0.825)	0.231 (0.573)	-0.451 (0.225)	-0.161 (0.701)
Ca^{2+}	-0.248 (0.543)	-0.258 (0.524)	-0.172 (0.678)	0.283 (0.468)	-0.033 (0.942)	0.056 (0.921)	-0.630 (0.062)	-0.181 (0.661)
Mg^{2+}	-0.246 (0.548)	-0.170 (0.682)	-0.054 (0.922)	0.310 (0.425)	-0.172 (0.678)	-0.148 (0.720)	-0.416 (0.284)	-0.305 (0.435)
Na^+	0.112 (0.795)	0.162 (0.699)	0.178 (0.670)	0.156 (0.704)	-0.097 (0.827)	-0.261 (0.516)	0.030 (0.948)	-0.158 (0.704)
CEC	0.456 (0.221)	0.573 (0.098)	0.481 (0.185)	0.045 (0.925)	-0.003 (0.994)	-0.319 (0.407)	0.646 (0.055)	-0.170 (0.682)
Sand	-0.081 (0.859)	-0.112 (0.795)	-0.018 (0.968)	0.318 (0.407)	-0.015 (0.974)	-0.018 (0.968)	-0.255 (0.528)	-0.210 (0.607)
Silt	0.027 (0.949)	0.048 (0.925)	-0.027 (0.949)	-0.376 (0.332)	-0.038 (0.932)	-0.126 (0.770)	0.335 (0.383)	0.208 (0.609)
Clay	0.175 (0.673)	0.205 (0.616)	0.162 (0.699)	0.287 (0.459)	0.189 (0.650)	0.520 (0.142)	-0.352 (0.367)	-0.046 (0.925)

LL, leaf length; LW, leaf width; LobN, number of lobes; PL, petiole length; 10LDW, dry weight of 10 leaves; MC, moisture contents; TH, tree height; RCD, root collar diameter; EC, electrical conductivity; OM, organic matter; TN, total nitrogen; AP, available phosphate; CEC, cation exchange capacity. [a] Pearson's correlation coefficients (*r*) are presented. Values in parentheses represent *p*-values adjusted using the False Discovery Rate (FDR) method. Asterisks denote statistical significance based on the adjusted *p*-value (** $p < 0.01$, * $p < 0.05$).

Table S7. Pearson's correlation coefficient between the growth characteristics of *Dendropanax trifidus* leaves and meteorological factors.

Meteorological Factors	Correlation coefficient (r)[a]							
	Growth traits							
	LL	LW	LobN	PL	10LDW	MC	TH	RCD
AAT	0.519<br>(0.142)	0.498<br>(0.166)	0.371<br>(0.338)	0.012<br>(0.976)	0.315<br>(0.415)	-0.385<br>(0.323)	0.562<br>(0.107)	-0.263<br>(0.513)
AAMT	0.546<br>(0.117)	0.453<br>(0.224)	0.295<br>(0.449)	0.093<br>(0.837)	0.393<br>(0.314)	-0.253<br>(0.531)	0.504<br>(0.161)	-0.108<br>(0.802)
AAmT	0.500<br>(0.166)	0.508<br>(0.153)	0.391<br>(0.314)	0.008<br>(0.983)	0.294<br>(0.449)	-0.407<br>(0.293)	0.554<br>(0.112)	-0.266<br>(0.507)
AMT	-0.058<br>(0.916)	-0.138<br>(0.744)	-0.137<br>(0.744)	0.115<br>(0.790)	0.244<br>(0.550)	0.313<br>(0.418)	-0.303<br>(0.438)	0.190<br>(0.650)
AmT	0.610<br>(0.073)	0.496<br>(0.169)	0.342<br>(0.380)	0.076<br>(0.867)	0.471<br>(0.195)	-0.318<br>(0.407)	0.407<br>(0.293)	-0.356<br>(0.363)
TP	0.348<br>(0.372)	0.350<br>(0.370)	0.303<br>(0.438)	0.215<br>(0.606)	0.053<br>(0.922)	-0.447<br>(0.229)	0.411<br>(0.293)	-0.321<br>(0.407)
RH	-0.262<br>(0.516)	-0.337<br>(0.382)	-0.288<br>(0.458)	0.002<br>(0.994)	-0.230<br>(0.575)	0.394<br>(0.314)	-0.399<br>(0.307)	0.287<br>(0.459)
SSH	-0.129<br>(0.762)	0.037<br>(0.934)	0.086<br>(0.851)	0.232<br>(0.573)	-0.201<br>(0.623)	0.269<br>(0.502)	0.052<br>(0.924)	0.408<br>(0.293)
ALT	0.330<br>(0.393)	**0.674***<br>**(0.039)**	**0.681***<br>**(0.036)**	0.123<br>(0.777)	-0.245<br>(0.549)	-0.164<br>(0.697)	**0.726***<br>**(0.016)**	-0.242<br>(0.557)

LL, leaf length; LW, leaf width; LobN, number of lobes; PL, petiole length; 10LDW, dry weight of 10 leaves; MC, moisture contents; TH, tree height; RCD, root collar diameter; AAT, annual average temperature; AAMT, annual average maximum temperature; AAmT, annual average minimum temperature; AMT, annual maximum temperature; AmT, annual minimum temperature; TP, total precipitation; RH, relative humidity; SSH, sum of sunshine hour; ALT, altitude. [a] Pearson's correlation coefficients (r) are presented. Values in parentheses represent p-values adjusted using the False Discovery Rate (FDR) method. Asterisks denote statistical significance based on the adjusted p-value (** $p < 0.01$, * $p < 0.05$).

Table S8. Pearson’s correlation coefficient between major compounds and the growth characteristics of *Dendropanax trifidus* leaves.

Growth traits	Correlation coefficient (r)[a]		
	Major compounds		
	CGA	Rutin	Total
LL	-0.033 (0.942)	0.150 (0.713)	0.103 (0.811)
LW	-0.292 (0.450)	-0.204 (0.617)	-0.235 (0.567)
LobN	-0.336 (0.383)	-0.299 (0.443)	-0.318 (0.407)
PL	-0.084 (0.854)	0.117 (0.787)	0.064 (0.902)
10LDW	0.269 (0.502)	0.556 (0.112)	0.491 (0.174)
MC	0.343 (0.380)	0.355 (0.364)	0.362 (0.351)
TH	-0.354 (0.365)	-0.519 (0.142)	-0.487 (0.177)
RCD	0.474 (0.191)	0.213 (0.606)	0.293 (0.450)

CGA, chlorogenic acid; LL, leaf length; LW, leaf width; LobN, number of lobes; PL, petiole length; 10LDW, dry weight of 10 leaves; MC, moisture contents; TH, tree height; RCD, root collar diameter.
[a] Pearson's correlation coefficients (r) are presented. Values in parentheses represent p-values adjusted using the False Discovery Rate (FDR) method. Asterisks denote statistical significance based on the adjusted p-value (** $p < 0.01$, * $p < 0.05$).

Table S9. Geographic information about the collection sites where leaves of *Dendropanax trifidus* were collected in South Korea, including the corresponding meteorological stations and distances.

Collection sites	Name of sites	ALT (m)	Latitude (°N)	Longitude (°E)	Meteorological station	Distance (km)
1	Geoje-si	67.8	34°56′xx.xx″	128°40′xx.xx″	Geoje	9.04
2	Namhae-gun	115.2	34°53′xx.xx″	127°51′xx.xx″	Namhae	10.33
3	Boseong-gun1	74.0	34°46′xx.xx″	127°08′xx.xx″	Boseong-gun	7.16
4	Boseong-gun2	30.2	34°51′xx.xx″	127°19′xx.xx″	Boseong-gun	14.19
5	Shinan-gun	33.7	34°50′xx.xx″	126°21′xx.xx″	Mokpo	3.59
6	Wando-gun	25.0	34°22′xx.xx″	126°38′xx.xx″	Wando	5.94
7	Jangseong-gun	197.8	35°25′xx.xx″	126°46′xx.xx″	Gochang-gun	7.42
8	Jindo-gun	23.0	34°26′xx.xx″	126°11′xx.xx″	Jindo-gun	6.54
9	Haenam-gun	91.8	34°36′xx.xx″	126°39′xx.xx″	Haenam	10.82
10	Seogwipo-si1 (Cul)	239.0	33°16′xx.xx″	126°27′xx.xx″	Seogwipo	10.96
11	Seogwipo-si2 (Cul)	10.0	33°15′xx.xx″	126°37′xx.xx″	Seogwipo	5.84
12	Seogwipo-si3 (Cul)	183.9	33°19′xx.xx″	126°42′xx.xx″	Seogwipo	15.87
13	Jeju-si1	214.0	33°27′xx.xx″	126°46′xx.xx″	Seongsan	12.65
14	Jeju-si2	104.2	33°29′xx.xx″	126°33′xx.xx″	Jeju	3.68
15	Seogwipo-si1 (Hab)	308.0	33°18′xx.xx″	126°35′xx.xx″	Seogwipo	8.37
16	Seogwipo-si2 (Hab)	535.4	33°21′xx.xx″	126°38′xx.xx″	Seogwipo	14.52
17	Seogwipo-si3 (Hab)	534.7	33°18′xx.xx″	126°33′xx.xx″	Seogwipo	6.9

ALT, altitude; Cul, cultivation site; Hab, habitat. Meteorological data for 2024 were obtained from 11 Automated Synoptic Observing System (ASOS) stations operated by the Korea Meteorological Administration (KMA). Distances represent straight-line distances between sampling sites and their assigned meteorological stations, calculated from geographic coordinates.
